# The influence of signal strength on conscious and nonconscious neural processing of emotional faces

**DOI:** 10.1093/nc/niaf001

**Published:** 2025-02-05

**Authors:** Insa Schlossmacher, Marie Herbig, Torge Dellert, Thomas Straube, Maximilian Bruchmann

**Affiliations:** Institute of Medical Psychology and Systems Neuroscience, University of Münster, von-Esmarch-Str. 52, Münster 48149, Germany; Otto Creutzfeldt Center for Cognitive and Behavioral Neuroscience, University of Münster, Fliednerstr. 21, Münster 48149, Germany; Institute of Medical Psychology and Systems Neuroscience, University of Münster, von-Esmarch-Str. 52, Münster 48149, Germany; Institute of Medical Psychology and Systems Neuroscience, University of Münster, von-Esmarch-Str. 52, Münster 48149, Germany; Otto Creutzfeldt Center for Cognitive and Behavioral Neuroscience, University of Münster, Fliednerstr. 21, Münster 48149, Germany; Institute of Medical Psychology and Systems Neuroscience, University of Münster, von-Esmarch-Str. 52, Münster 48149, Germany; Otto Creutzfeldt Center for Cognitive and Behavioral Neuroscience, University of Münster, Fliednerstr. 21, Münster 48149, Germany; Institute of Medical Psychology and Systems Neuroscience, University of Münster, von-Esmarch-Str. 52, Münster 48149, Germany; Otto Creutzfeldt Center for Cognitive and Behavioral Neuroscience, University of Münster, Fliednerstr. 21, Münster 48149, Germany

**Keywords:** continuous flash suppression, contrast, EEG, facial expression, nonconscious processing, stimulus strength

## Abstract

Consciously perceived emotional relative to neutral facial expressions evoke stronger early and late event-related potential (ERP) components. However, the extent of nonconscious neural processing of emotional information in faces is still a matter of debate. One possible reason for conflicting findings might relate to threshold effects depending on the sensory strength of stimuli. In the current study, we investigated this issue by manipulating the contrast of fearful and neutral faces presented with or without continuous flash suppression (CFS). Low, medium, and high contrasts were calibrated individually so that faces were consciously perceived at all contrast levels if presented without CFS. With CFS, however, low- and medium-contrast faces remained nonconscious, while high-contrast faces broke the suppression. Without CFS, ERPs showed an increased early negativity and late positivity in response to fearful vs. neutral faces regardless of contrast. Under CFS, we observed differential early negativities for suppression-breaking high-contrast fearful vs. neutral faces. For nonconscious faces, however, the contrast level modulated the difference between fearful and neutral faces, showing enhanced early negativities only at medium contrast and an inverted effect at low contrast. Additional analysis of late positivities provided evidence for the absence of an effect at low and medium contrast, while at high-contrast, fearful faces elicited a larger positivity than neutral ones. Taken together, our findings demonstrate the significance of stimulus strength for nonconscious emotion processing under CFS, implying that early negative ERP differences between neutral and fearful faces depend on stimulus contrast near the detection threshold.

## Introduction

Several electrophysiological studies show increased event-related potentials (ERPs) in response to fearful compared to neutral faces, even if they are not consciously perceived (Liddell et al. 2004, Eimer et al. 2008, Kiss and Eimer 2008, Pegna et al. 2008, 2011, Jiang et al. 2009, Smith 2012, Zotto and Pegna 2015, Doradzińska and Bola 2023), which is classically interpreted as a result of highly automatized processing of stimuli with biological significance (for a review, see Tamietto and de Gelder 2010).

However, upon closer inspection, the results of these studies are heterogeneous, with several studies reporting null findings (Schlossmacher et al. 2017, Qiu et al. 2022, 2022a, 2022b, 2023). Correspondingly, a recent review concluded that, as of yet, no consensus regarding the existence or scope of nonconscious emotion processing has been reached (Mudrik and Deouell 2022). The heterogeneity of results can partly be attributed to the variety of stimuli and experimental paradigms (Tamietto and de Gelder 2010, Axelrod et al. 2015), but even among highly comparable studies, we find apparently contradicting results (Jiang et al. 2009, Schlossmacher et al. 2017).

In the present study, we focus on continuous flash suppression (CFS), a “blinding” technique relying on a binocular setup (Tsuchiya and Koch 2005). The stimuli of interest, e.g. faces, are presented to one eye, while flickering so-called “Mondrian” images are presented to the other. In this setup, the rapidly changing Mondrian images lead to a relatively strong and long-lasting suppression of the target stimuli. It should be noted, however, that the strength of suppression is tightly connected to the stimulus strength, e.g. contrast. The so-called breaking CFS paradigm (b-CFS; Stein 2019) makes use of the fact that initially invisible stimuli can emerge from suppression when their contrast is increased and infers differential nonconscious processing where detection times diverge.

In the past, while one ERP study found that CF-suppressed fearful faces elicited a stronger N170 response than suppressed neutral faces (Jiang et al. 2009), our group found evidence for the absence of such effects in a highly similar study that employed a more rigorous control for stimulus breakthrough by individually reducing stimulus contrast (Schlossmacher et al. 2017).

**Figure 1:**
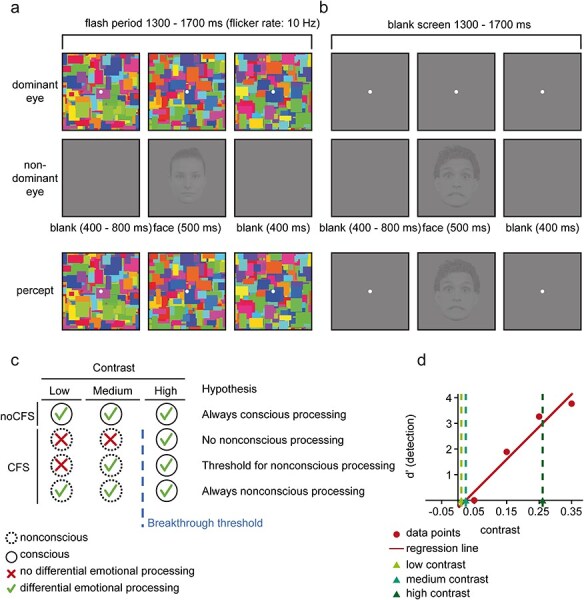
Schematic illustration of CFS and noCFS trials, the two-threshold account, and the contrast estimation. (a) CFS condition of the main experiment (identical setup used for contrast calibration). In the contrast calibration, the face contrast varied, while in the main experiment, it was stable at the determined contrast levels. The low- and medium-contrast conditions were designed so that participants should perceive the flickering Mondrian images only but not the face stimulus. (b) noCFS condition of the main experiment. In this condition, participants were meant to consciously perceive the face stimulus. Example face stimuli taken from the Radboud Faces Database (Langner et al. 2010). (c) The nonconscious threshold hypothesis with a specific contrast threshold for ERP effects during nonconscious processing of emotional and neutral faces. (d) Example of contrast calibration results for one participant.

We have previously proposed a threshold hypothesis for nonconscious emotion processing (Schlossmacher et al. 2017) to reconcile seemingly contradicting findings. This account has also been implied in previous studies, claiming in one way or another that intermediate degrees of stimulus strength may exist where conscious perception is not yet possible, but some form of differentiation between stimulus categories is (Peremen and Lamy 2014, Sterzer et al. 2014). From this point of view, there might be a threshold contrast at which nonconscious differential processing of fearful and neutral facial expressions becomes possible (see Fig. 1c for a schematic representation). Below this threshold, no differential effects for fearful compared to neutral faces are observed. While this threshold might be hard to find in practice, e.g. due to noise in measuring the awareness threshold, it should be possible to show that stimulus strength, i.e. contrast, plays a role in nonconscious emotional processing. Varying the contrast of nonconscious face stimuli using CFS may then yield three theoretically plausible results as illustrated in Fig. 1c, where “high contrast” refers to a contrast high enough to break CFS, thus enabling conscious perception. “Medium” and “low contrast” refer to contrasts just below and considerably below the detection threshold under CFS, respectively. Specifically, medium contrast constitutes the highest possible contrast at which stimuli are still suppressed from consciousness by CFS. Without CFS, stimuli are consciously perceived at all three contrast levels.

According to the threshold account mentioned earlier, the medium-contrast condition allows for differential nonconscious facial expression processing, while low-contrast stimuli are insufficient for nonconscious processing. Observing evidence for differential processing in both nonconscious conditions (i.e. at medium and low contrast) would contradict this hypothesis and rather imply that every stimulus consciously perceived and differentially processed without CFS does also elicit differential processing while suppressed from consciousness with CFS. In a third scenario, no nonconscious processing is possible under CFS at all.

Full-contrast, unsuppressed fearful compared to neutral faces typically produce at least three spatiotemporally discernible effects on ERPs (for reviews, see Eimer and Holmes 2007, Hinojosa et al. 2015, Schindler and Bublatzky 2020). Firstly, the N170, a negative deflection of the ERP observed around 170 ms after stimulus onset at occipito-temporal channels (see Hinojosa et al. 2015), is reliably enhanced by fearful faces, irrespective of the attentional focus or the participants’ task (Rellecke et al. 2011, Neath-Tavares and Itier 2016, Schindler et al. 2020, 2021a, 2021b, 2022c). Secondly, the early posterior negativity (EPN), which typically peaks between 200 and 300 ms, has frequently been found to be increased for fearful compared to neutral facial expressions (Walentowska and Wronka 2012, Wieser et al. 2012, Schindler et al. 2019, Durston and Itier 2021), but depends on attentional resources (Schindler et al. 2020, 2022a). Thirdly, the late positive potential (LPP), a parieto-central positive component peaking between 400 and 800 ms, is also enhanced for fearful faces, but only if the facial expression itself is task-relevant (Schindler et al. 2020, 2021b, 2022b).

However, with low-contrast faces, one study found that the peak of N170 is delayed, and difference waves between fearful and neutral faces no longer produce two temporally or topographically discernible peaks for the N170 and the EPN, even if presented without CFS (Schlossmacher et al. 2017). In the context of the present study, we therefore refrain from differentiating between the N170 and the EPN and summarize them under the term “early negativities.” The initially mentioned heterogeneity of results concerning neural correlates of nonconscious emotion processing pertains to these early negativities since several studies have shown that later components like the LPP critically depend on conscious perception (Kiss and Eimer 2008, Pegna et al. 2008, Smith 2012).

With the present study, we sought to clarify whether nonconscious processing of fearful vs. neutral faces under CFS as indexed by differential early negativities depends on stimulus contrast. We hypothesized that fearful expressions enhance early negativities if presented without CFS across all contrast conditions. With CFS, we expected facial expression effects only in the high- and medium-contrast conditions. Furthermore, we hypothesized that fearful faces enhance LPP amplitudes in all conscious conditions but not in the nonconscious conditions (low and medium contrast with CFS).

## Methods

### Participants

The sample size was based on our previous study (required *N* = 45; Schlossmacher et al. 2017), where we did an *a priori* power calculation using G*Power 3.1.9.2 (Faul et al. 2007) based on the *F*-value reported in the most comparable study by Jiang et al. (2009). This led to the calculated optimal sample size of *N* = 45. In order to achieve at least this sample size while compensating for an expected dropout rate of 10%–20%, we collected data from 62 participants. Participants were excluded if their contrast calibration was unsuccessful, as indicated by a high performance in the detection task (*d'* > 1, *N* = 9), and if their electroencephalography (EEG) data quality was insufficient (extremely low signal-to-noise ratio as judged by visual inspection, *N* = 5).

The final sample size amounted to 48 participants (37 females, 11 males) aged 18–40 years (*M* = 23.94, SD = 5.02). All participants had normal or corrected-to-normal vision and were right-handed. Participants volunteered and were compensated with 10 € per hour. Before starting, participants were given written instructions on the experimental task and had the opportunity to ask further questions. The study was approved by the local ethics committee.

### Apparatus

The experiment was run using MATLAB and the Psychophysics Toolbox (Brainard 1997, Pelli 1997, Kleiner et al. 2007). A Sony PVM 2541 OLED monitor at 60 Hz with a resolution of 1920 × 1280 pixels was employed for stimulus display. The viewing distance amounted to 60 cm. To respond, participants used the numeric keypad of a standard keyboard (Keys 1 and 2). At all times, the monitor displayed two symmetrical gray rectangles of 7.5 × 10 degrees of visual angle (°), horizontally distanced 18° in front of a black background. Both rectangles were surrounded by an ornate frame to promote the convergence of the two images in the participants’ perception. A mirror stereoscope was installed in front of the screen in order to accomplish binocular fusion of the two rectangular images. To prevent head movements during the experiment, participants used a chin rest for stabilization.

### Stimulus material

Forty-eight faces from the Radboud Faces Database (Langner et al. 2010) served as stimuli for this experiment. Only faces with a frontal gaze direction shot at a camera angle of 90° were used. All faces were converted into grayscale images, cut to square format using Adobe Photoshop CS6 and presented on a middle gray (Red/Green/Blue): 126, 126, 126) background within the rectangular frames. Twenty-four identities (12 female) were chosen from the database, of which 12 were used in the contrast calibration and 12 in the main experiment to minimize possible habituation effects. Each identity featured a fearful and neutral expression and spanned 4.2 × 4.2°. We manipulated the contrast of the face stimuli by scaling the gray values symmetrically around the medium gray value. Contrast is therefore defined as the range of gray levels of an image irrespective of its spatial frequency. Mondrian images consisted of randomly colored rectangles (HSB color space with random hue H, full saturation S and full brightness B). For examples, see Fig. 1a.

### Experimental procedure

#### Eye dominance measurement

Eye dominance was measured using a variant of the Porta test (Porta 1593). Participants were instructed to extend their arm and to visually cover an object with their thumb; they were then instructed to alternately close one eye at a time. The nondominant eye was defined as the eye for which, when opened, the thumb was perceived as further away from the covered object, leading to a dichotomous classification of left or right eye dominance. For some participants, the Porta test did not yield conclusive results. For these participants, we used a modified version of the Area-Blackness-Composition test of eye dominance (Miles 1929). Participants were instructed to extend both arms and form a small opening by bringing their hands together. They were then instructed to fixate on an object ∼5 m away through the hole and alternately close their eyes. The nondominant eye was defined as the eye for which, when opened, the object was not inside the hole anymore. As it has been recommended for CFS (Yang et al. 2010), the nondominant eye—as defined by these procedures—was shown the face stimuli in the contrast calibration and the main experiment.

#### Contrast calibration

An individual contrast calibration was carried out to determine three different contrast levels (low, medium, and high) to be used in the main experiment. In this pretest, stimuli were always presented with CFS. We used the method of constant stimuli (Simpson 1988), presenting faces at four different contrast levels (5%, 15%, 25%, and 35% of maximal contrast). Additionally, catch trials without a face were included.

Each contrast calibration trial began with a fixation period of 100 ms, after which the presentation of Mondrian patterns at a flicker rate of 10 Hz to the dominant eye started. After 600 ms (±0–200 ms), face stimuli were presented to the nondominant eye for 500 ms. After the offset of the faces, the presentation of Mondrian patterns continued for 400 ms. After each trial, participants were asked to report their perception in a two-alternative forced choice (2AFC) task, pressing Key 1 if they only perceived the Mondrian patterns and Key 2 if they saw “anything else” besides the Mondrian patterns. Each condition was presented 48 times, amounting to 240 trials divided into four blocks by short breaks of 10 s every 60 trials. After completion, *d'* (Macmillan and Creelman 2005) was computed for each contrast level, and a regression line was fitted to the data points (see Fig. 1d). Based on this regression line, we extracted the values corresponding to a *d'* value of 0 (medium contrast) and a *d'* value of 3 (high contrast). The low contrast was computed by dividing the medium contrast by 2. These three values were then used during the main experiment. If the contrast calibration did not produce a satisfactory result, e.g. values below zero for the medium-contrast condition, the experimenter adjusted initial contrast values to match the estimated range of informative contrasts, and the procedure was repeated.

#### Main experiment

Twelve new faces from the Radboud Faces Database were used in the main experiment. Different identities with neutral and fearful facial expressions were used for the condition with CFS and the condition without CFS (in short: noCFS), resulting in six identities in each condition (three males and three females). Faces were presented at the three different contrast levels determined during the contrast calibration. Each identity was presented at all three contrast levels, featuring a fearful and neutral expression. The same identities were used for all participants. In addition, there were catch trials where no face was presented. All contrast and catch conditions were presented with and without CFS, and the order of presentation was randomized. The timing of the stimuli was identical to the contrast calibration (see Fig. 1a and b). A response prompt was presented only on a randomly chosen third of the trials to allow for more trials in the ERP analysis while keeping the experiment at a reasonable length. First, participants were asked the same question as in the contrast calibration, i.e. whether they only perceived the Mondrian patterns (Key 1) or anything else (Key 2). Second, they were asked to indicate or guess whether the face had a neutral (Key 1) or fearful (Key 2) facial expression. Each of the 14 conditions (2 emotions × 3 contrasts × 2 visibility conditions + 2 catch conditions) comprised 60 trials, resulting in 840 trials in total. Since participants were not informed ahead of a trial if a response prompt would follow, no modulation of attention or other task-related confounds between response and nonresponse trials was to be expected. Hence, we included all trials, irrespective of response prompt, in our preprocessing pipeline.

The experiment was divided into seven blocks of 120 trials, each separated by a self-paced break for the participants. In total, the experimental procedure took ∼1 h to complete. As we expected an increase in visibility as the experiment progressed, contrasts were continuously recalibrated during the breaks by computing a combined *d'* for the medium- and low-contrast condition of the preceding block of trials and reducing the contrasts of both the low and medium condition by 20% if *d'* exceeded 0.3. This threshold was chosen based on theoretical and practical considerations: first, while objective measures have the advantage of providing criterion-controlled performance measures, they may provide an overly conservative estimate of the awareness threshold, as nonconscious processing may contribute to above chance-level performance (Merikle 1982, Schmidt and Vorberg 2006). Thus, we regarded *d'* > 0 as an overly conservative recalibration criterion. Secondly, due to the unreliability of estimating *d'* for single blocks, we chose this value to (i) reduce contrast for participants who might experience perceptual learning and (ii) not to reduce contrast for participants too much when only random fluctuations led to *d'* > 0.

### EEG recording and preprocessing

A 256-channel HydroCel Geodesic Sensor Net (Electrical Geodesic Inc., Eugene, OR) was applied, with each electrode consisting of a silver chloride-plated carbon-fiber pellet surrounded by a sponge. The electrode Cz was placed according to the 10/20 system and used as an online reference. The ground electrode was positioned posterior to Cz. Electrical potentials were recorded with a sampling rate of 500 Hz using a NetAmps 400 amplifier. Impedances were held below 100 kΩ (a recommended value for the high-impedance system used here). A built-in analog antialiasing low-pass filter at 200 Hz and a high-pass filter at 0.1 Hz were applied before digitization. Saved data were exported to European Data Format and preprocessed in BESA Research 6.0. First, data were re-referenced to average reference. Eye-movement artifacts were corrected using the automatic eye-artifact correction method implemented in BESA (Ille et al. 2002). Filtered data were segmented from 200 ms before stimulus onset until 600 ms after stimulus onset. The remaining artifacts were rejected based on an absolute threshold chosen manually based on data quality (e.g. <120 μV), signal gradient (< 5 μV/∂T), and low signal (i.e. the SD of the gradient, >0.01 μV/∂T). Noisy electrodes were interpolated using a spline interpolation procedure, and trials were baseline-corrected using the 200 ms before stimulus onset. Finally, averaged data were exported to MATLAB for statistical analysis (see below).

### Statistical analysis

Statistical analysis employed a factorial mass univariate approach in combination with cluster-based permutation (CBP) tests (Fields and Kuperberg 2020). We included the factors facial expression and contrast in a 2 × 3 design separately for the CFS and the noCFS conditions, i.e. we tested for a main effect of facial expression, a main effect of contrast, and an interaction of facial expression and contrast. In order to enhance the power of the cluster-based analysis, we chose specific time intervals and electrode locations based on previous research. For the analysis of the early negativities, we chose a time interval from 150 to 300 ms in posterior electrodes (Hajcak et al. 2012, Schindler and Bublatzky 2020; see Figs 4 and 5). For the LPP, we selected central electrodes and an interval from 300 to 600 ms (Hajcak et al. 2012, Schindler and Bublatzky 2020; see Figs 4 and 5). This interval was based on Schlossmacher et al. (2017)  *a priori*. Since this interval is short compared to other studies on the LPP (Schindler et al. 2020, 2021b, 2022b), we additionally analyzed a longer interval between 300 and 800 ms, which yielded the same results (see the Supplementary data). Clusters were formed by two or more neighboring sensors (in time and space) whenever the *F*-values exceeded the cluster threshold (*α* = 0.05). The cluster mass, sum(*F*), was calculated by adding all *F*-values within a cluster. The number of permutations was set to 5000, and the significance value for testing the null hypothesis amounted to *α* = 0.05. Prior to the analysis, ERPs were down-sampled to 125 Hz and low-pass filtered at 25 Hz (roll-off: −24 dB/octave) to further enhance statistical power (Luck 2005). We multiplied the resulting *P*-values by two in order to correct for multiple CBP tests (one for each time window). After applying the CBP approach, cluster averages from significant clusters were computed and used to investigate significant interaction effects. Then, we used *t*-tests with the Bonferroni–Holm correction method to compare cluster averages.

Task performance quantified as *d'* (Macmillan and Creelman 2005) was computed for the detection and the discrimination task. Cluster averages and *d'* were analyzed using repeated-measures Analysis of Variance (ANOVAs) and *t*-tests. Whenever sphericity was violated, the Greenhouse–Geisser correction was applied, and corrected *P*-values as well as $\hat \varepsilon $-values are reported later. As some of our conclusions rely on null effects, we additionally report Bayes factors (BF), with BF_01_ denoting the evidence for the null hypothesis and BF_10_ for the alternative hypothesis. We use the conventions from the study by Jeffreys (1961) to interpret the results of our Bayesian analyses. All CBPs were done using the Factorial Mass Univariate Toolbox (FMUT; Fields 2017). Other statistical tests relied on the statistics program R (R Core Team 2023).

## Results

**Figure 2:**
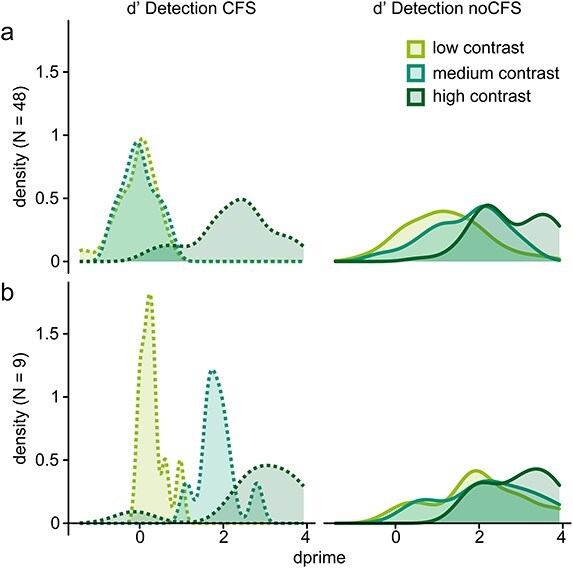
Densities of *d'* detection values for (a) participants included in the analysis (*N* = 48) and (b) participants excluded because of *d'* > 1.

Five participants had to be excluded due to insufficient EEG data quality, and nine due to a failure of the contrast calibration and unsuccessful recalibration leading to a high performance (*d'* > 1) in the medium-contrast condition during CFS (see Fig. 2).

With regard to the remaining 48 participants, an average of 18.08 (SD = 6.46) electrodes were interpolated, corresponding to 7.06% (SD = 2.52%) of the total of 256 electrodes. The artifact rejection threshold amounted to 113.48 (SD = 24.94) µV, resulting in the rejection of an average of 22.45% (SD = 15.08%) of trials. This high percentage resulted from a large amount of eye movements and blink artifacts due to the experimental setup with the stereoscope and increased noise due to the strenuous task. Out of 48 participants, 14 were classified as left-eye-dominant. Average contrasts used in the main experiment amounted to 0.017 (SD = 0.012) in the low-contrast condition, 0.032 (SD = 0.019) in the medium-contrast condition, and 0.46 (SD = 0.27) in the high-contrast condition.

### Behavioral data

**Figure 3:**
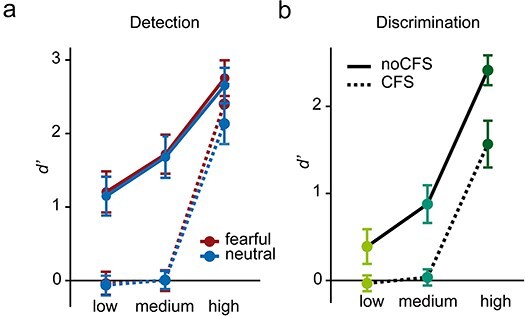
Behavioral data. (a) Detection performance measured by *d'*. (b) Discrimination performance measured by *d'*. Error bars depict the standard error of the mean.

In the detection task, the 2 × 2 × 3 repeated-measures ANOVA of *d'* with the factors emotion, CFS, and contrast indicated a significant main effect of emotion [*F*(1,47) = 8.11, *P* = .007], CFS [*F*(1,47) = 109.88, *P* < .001], and contrast [*F*(2,94) = 270.56, *P* < .001, $\hat \varepsilon $ = 0.63] as well as significant interactions between emotion and contrast [*F*(2,94) = 4.60, *P* = .01] and between CFS and contrast [*F*(2,94) = 30.69, *P* < .001, $\hat \varepsilon $ = 0.81], see Fig. 3a. Without CFS, *d'* increased from low to medium to high contrast (*P* < .001, BF_10_ > 30). With CFS, *d'* differed significantly between the high contrast and both low and medium contrast (*P* < .001, BF_10_ > 30) but not between the low- and medium-contrast condition [fearful: *t*(47) = −64, *P* = .53, BF_01_ = 5.27; neutral: *t*(47) = −0.90 *P* = .37, BF_01_ = 4.35]. Importantly, BFs testing the null hypothesis in the low- and medium-contrast conditions with CFS indicated evidence for the absence of an effect [fearful_low_: *t*(47) = −0.56, *P* = .57, BF_01_ = 5.48; fearful_medium_: *t*(47) = 0.02, *P* = .99, BF_01_ = 6.38; neutral_low_: *t*(47) = −0.88, *P* = .38, BF_01_ = 4.42; neutral_medium_: *t*(47) = 0.10, *P* = .92, BF_01_ = 6.35], while all other conditions differed significantly from zero (*P* < .001, BF_10_ > 30). Fearful faces were significantly better detected than neutral ones in the high-contrast condition with and without CFS [with CFS: *t*(47) = −4.22, *P* < .001, BF_10_ = 210.86; without CFS: *t*(47) = −2.25, *P* = .03, BF_10_ = 1.53], while in all other conditions, there was evidence for the absence of an effect (*P* > .05, BF_01_ > 3). Furthermore, *d'* computed after collapsing across low and medium contrasts showed evidence for the absence of an effect for both neutral [*t*(47) = −0.10, *P* = .92, BF_01_ = 6.35] and fearful faces [*t*(47) = −0.04, *P* = .97, BF_01_ = 6.37].

In the discrimination task, the 2 × 3 repeated-measures ANOVA of *d'* with the factors contrast and CFS indicated a significant main effect of contrast [*F*(2,94) = 352.77, *P* < .001, $\hat \varepsilon $ = 0.79], CFS [*F*(1,47) = 129.37, *P* < .001] as well as a significant interaction [*F*(2,94) = 4.81, *P* = .02, $\hat \varepsilon $ = 0.76]; see Fig. 3b. Without CFS, *d'* increased from the low- to the medium- to high-contrast condition (*P* < .001, BF_10_ > 30). With CFS, *d'* differed significantly between the high contrast and both low and medium contrast (*P* < .001, BF_10_ > 30) but not between the low- and medium-contrast condition [*t*(47) = −1.23, *P* = .22, BF_01_ = 3.14]. Importantly, BFs testing the null hypothesis in the low- and medium-contrast conditions with CFS indicated evidence for the absence of an effect [low: *t*(47) = −0.72, *P* = .48, BF_01_ = 5.00; medium: *t*(47) = 0.76, *P* = .45, BF_01_ = 4.85], while all other conditions differed significantly from zero (*P* < .001, BF_10_ > 30). Furthermore, collapsing across low- and medium-contrast conditions showed evidence for the absence of an effect [*t*(47) = 0.48, *P* = .63, BF_01_ = 5.72].

### EEG data

#### Early negativities

**Figure 4:**
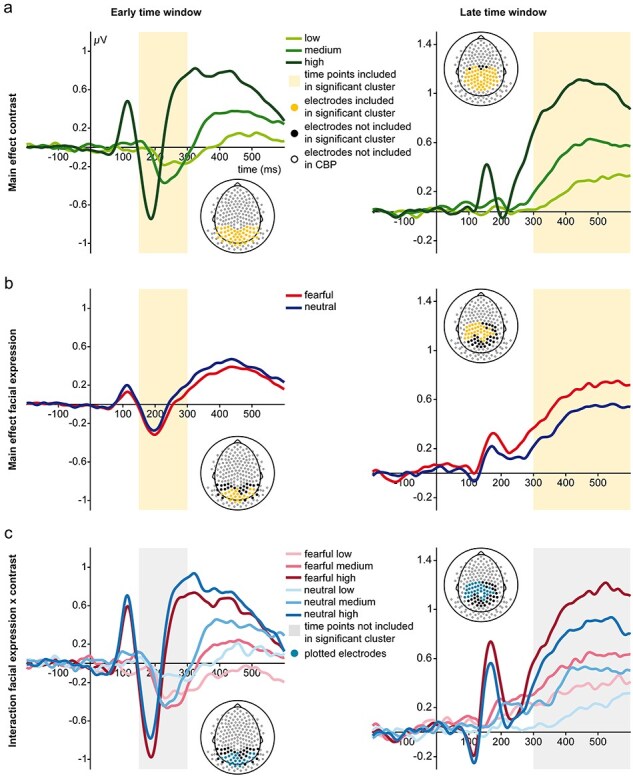
FMUT results for noCFS conditions. (a) Main effect contrast in the early and late time window. (b) Main effect facial expression in the early and late time window. (c) Interaction effect in the early and late time window. Note that the interaction effect is also depicted for reasons of consistency. As the test of the interaction did not yield a significant cluster, we used electrodes and time window of the main effect facial expression obtained in the noCFS condition in the early and late time window.

Without CFS, CBPs revealed a significant main effect of contrast [sum(*F*) = 10 333.23, *P* < .001] extending from 150 to 300 ms with a temporal peak at 260 ms. Furthermore, we found a significant main effect of facial expression [sum(*F*) = 976.52, *P* = .01] extending from 150 to 300 ms with a temporal peak at 236 ms. No significant interaction was found (all clusters uncorrected *Ps* > .45). Further inspection of the main effect of facial expression revealed a stronger negativity for fearful compared to neutral faces. See the left column in Figs 4 and 6a for a visualization of the results.

**Figure 5:**
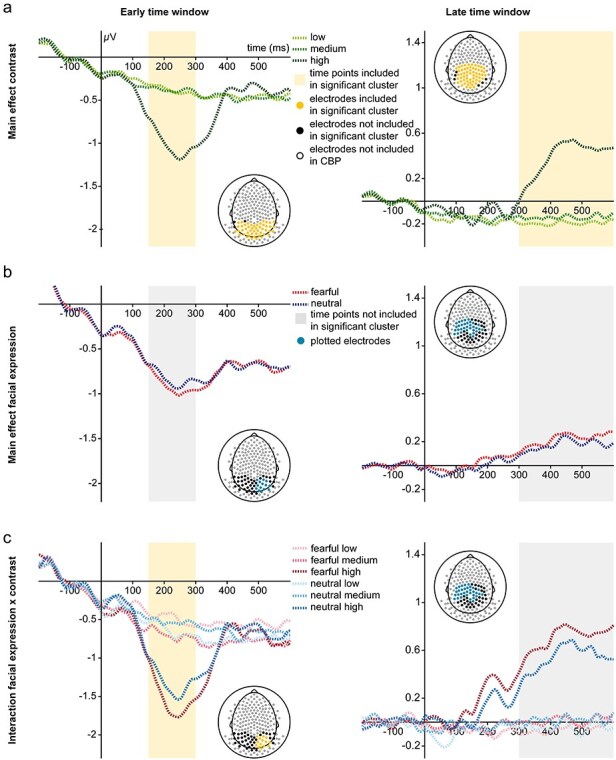
FMUT results in CFS conditions. (a) Main effect contrast in the early and late time window. (b) Main effect facial expression in the early and late time window. (c) Interaction of facial expression and contrast in the early time window. Note that effects and time windows with no significant clusters are also depicted for reasons of consistency. For the main effect of facial expression in the early time window, electrodes and time window of the significant interaction was used. As in the later time window neither the main effect of emotion nor the interaction yielded a significant cluster, we used electrodes and time window of the main effect facial expression obtained in the noCFS condition.

**Figure 6:**
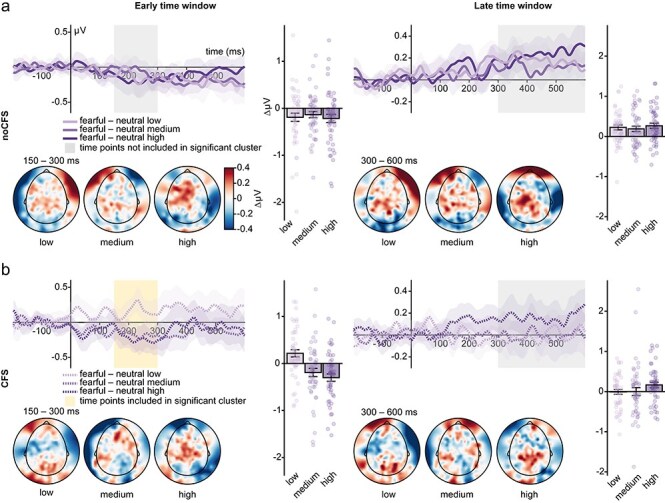
Difference waves, topographies, and bar plots of cluster averages for early and late time windows in the CFS and noCFS condition. (a) Early and late difference waves (fearful—neutral) for each contrast in the noCFS condition. Time interval of topographies and electrode selection rely on the significant facial expression main effect. (b) Early and late difference waves (fearful—neutral) for each contrast in the CFS condition. In the early time interval, time interval of topographies and electrode selection for waveforms rely on the significant interaction effect of facial expression × contrast. In the late time interval, time interval of topographies and electrode selection for waveforms rely on the significant facial expression main effect in the noCFS condition. Error bars depict the standard error of the mean. Dots represent single-subject cluster averages.

With CFS, CBPs revealed a significant main effect of contrast [sum(*F*) = 13 248.15, *P* < .001] extending from 150 to 300 ms with a temporal peak at 212 ms. We found no significant main effect of facial expression (all clusters uncorrected *P*s > .40), while a significant cluster for the Contrast × Facial Expression interaction was found [sum(*F*) = 655.99, *P* = .03]. The significant cluster spanned 150 to 300 ms (temporal peak 220 ms) and was lateralized to right electrodes. Further testing the interaction on extracted cluster averages revealed significant effects of facial expression depending on contrast. Cluster averages of expression effect differences (fearful minus neutral) for high and medium contrast differed significantly from low-contrast expression differences [low vs. high: *t*(94) = 4.71, *P*_holm_ < .001, BF_10_ = 2011.79; low vs. medium: *t*(94) = 3.50, *P*_holm_ = .001, BF_10_ = 39.31], while evidence for the absence of an effect between high and medium contrast was obtained [medium vs. high: *t*(94) = 0.92, *P*_holm_ = 0.36, BF_01_ = 3.20]. Furthermore, testing these cluster averages against zero revealed a stronger negativity for fearful compared to neutral faces in the high- and medium-contrast condition [high: *t*(47) = −3.83, *P*_holm_ = .001, BF_10_ = 69.04; medium: *t*(47) = −2.18, *P*_holm_ = .03, BF_10_ = 1.35] and a stronger positivity in the low-contrast condition [*t*(47) = 2.82, *P*_holm_ = 0.01, BF_10_ = 5.22]. See the left column in Figs 5 and 6b for a visualization of the results.

#### Late positivities

Without CFS, CBPs revealed a significant main effect of contrast [sum(*F*) = 29 229.46, *P* < .001] including time points from 300 to 600 ms with a temporal peak at 324 ms. Furthermore, a significant main effect of facial expression [sum(*F*) = 3930.07, *P* = .003] extending from 300 to 600 ms with a temporal peak at 564 ms was found. No significant interaction was found (all clusters uncorrected *Ps* > .16). Further inspection of the main effect of facial expression revealed a stronger positivity for fearful compared to neutral faces. See the right column in Figs 4 and 6a for a visualization of the results.

With CFS, CBPs revealed a significant main effect of contrast [sum(*F*) = 29 907.53, *P* < .001] including time points from 300 to 600 ms with a temporal peak at 428 ms. No significant main effect of facial expression (all clusters uncorrected *Ps* > .14) and no significant interaction (all clusters uncorrected *Ps* > .25) were found. As neither the main effect of facial expression nor the interaction revealed significant clusters, we wanted to investigate whether we could obtain evidence for the absence of an effect. Therefore, we extracted cluster averages based on the significant main effect of facial expression in the condition without CFS. Testing these cluster averages against zero revealed evidence for the absence of an effect in the low- and medium-contrast conditions [low: *t*(47) = −0.11, *P*_holm_ = 1, BF_01_ = 6.34; medium: *t*(47) = 0.008, *P*_holm_ = 1, BF_01_ = 6.38], while in the high-contrast condition, fearful expressions elicited a stronger positivity than neutral expressions [*t*(47) = 2.54, *P*_holm_ = .04, BF_10_ = 2.79]. See the right column in Figs 5 and 6b for a visualization of the results.

In order to test whether residual awareness could, in principle, explain the results obtained in the CFS condition, we tested correlations of cluster averages with average detection *d'* values, hit rates, and *d'* collapsed across low and medium contrast in an exploratory analysis. For the detection *d'* and early negativity cluster averages, no correlations reached significance (*P*s > .05; −0.24 < *r* < −0.01), and evidence for a null effect remained inconclusive (low: BF_01_ = 0.92; high: BF_01_ = 1.22) in all but the medium-contrast condition (BF_01_ = 3.07). Regarding the hit rate from the detection task and early negativity cluster averages, no correlations reached significance (*P*s > .05; −0.15 < *r* < 0.04), and evidence for a null effect remained inconclusive (low: BF_01_ = 2.79; medium: BF_01_ = 2.96; high: BF_01_ = 1.88). For the collapsed detection *d'* and early negativity cluster averages, no correlations reached significance (*P*s > .05; −0.22 < *r* < 0.18), and evidence for a null effect remained inconclusive (low: BF_01_ = 1.15; medium: BF_01_ = 1.55).

## Discussion

In this study, we investigated the influence of stimulus contrast on differential emotional processing in conscious and nonconscious conditions with and without CFS. Behavioral data indicated that we successfully manipulated awareness, as indexed by substantial evidence against detection and discrimination above chance in the two conditions that were designed to remain nonconscious (low and medium contrast with CFS). Without CFS, we found facial expression effects on early and late ERP components in all contrast conditions. With CFS, both the consciously perceived high-contrast stimuli and the unaware medium-contrast stimuli elicited an increased early negativity in response to fearful vs. neutral faces, whereas only high-contrast faces showed some evidence of a late positivity.

In the Introduction, we proposed a threshold hypothesis of nonconscious expression effects under CFS influenced by stimulus strength (Fig. 1c). However, while we indeed found effects of stimulus contrast, we report a differential pattern of results in both nonconscious conditions. At medium contrast, we observed effects of nonconscious facial expressions similar to those at high contrast. On the contrary, at low contrast, we observed an inverted effect. Similar inverted effects in nonconscious conditions have been reported in other studies on face processing using CFS (Geng et al. 2012) and the attentional blink (Eiserbeck et al. 2024). While we can only speculate about the reason for an inversion of the effect, we conclude that contrast drastically modulates the results in nonconscious conditions. The presence of the inverted effect at low contrast challenges the threshold hypothesis proposed in Fig. 1c, according to which only a medium contrast (i.e. a contrast just below the awareness threshold) should yield differential expression effects, while a lower contrast should not. Instead, our results rather point to a hypothesis in which all stimuli that are consciously perceived and differentially processed without CFS are also nonconsciously processed to some extent with CFS depending on contrast (bottom hypothesis in Fig. 1c), albeit with inverted effects at low contrast. Nonetheless, the medium contrast chosen here might have corresponded to a sweet spot where nonconscious emotion processing resembles conscious emotion processing. The current results thus highlight the importance of carefully considering the contrast adjustment in studies of nonconscious processing.

An alternative explanation for emotion-selective responses during CFS is the partial awareness hypothesis (Kouider and Dupoux 2004, Kouider et al. 2010). This theory suggests that a combination of unsuccessfully suppressed stimulus features, top-down expectations, and context clues can give rise to semantic processing outside of global stimulus perception. This might then suggest nonconscious processing when subjects have actually been aware of some fragments of the suppressed stimulus. This explanation might initially seem to be plausible, especially since CFS has previously been reported to allow breakthroughs of stimulus fragments and low-level stimulus features (Gelbard-Sagiv et al. 2016, Moors et al. 2017). In line with this, a CFS study on identity priming of faces showed that high-level priming effects were observed only when low-level features were consciously perceived (Gelbard-Sagiv et al. 2016). However, our behavioral data show that under CFS at low and medium contrast, both the neutral and fearful faces were completely invisible to the participants. This was shown by evidence for the absence of above chance-level performance in both the detection and the discrimination task. Furthermore, correlations between cluster averages and *d'* as well as hit rates remained insignificant. Still, it should be taken into account that the number of trials that went into the estimation of *d'* might have been too low to get a reliable estimate (Yaron et al. 2024). Furthermore, using a correlation between ERP effects and *d'* in order to investigate residual awareness has been criticized due to correlation attenuation stemming from the low reliability of the measures (Malejka et al. 2021). While collapsing across the low- and medium-contrast conditions to improve the measure’s reliability confirmed our previous analysis, the results of the correlational analysis should be interpreted with care. Thus, in the current study, partial awareness cannot be completely excluded, although it seems highly unlikely given the behavioral results confirming a chance-level performance.

Taken together, our findings reconcile the diverging findings of previous ERP research, in which some studies indicated early emotion effects in nonconscious conditions (Jiang et al. 2009), while others found evidence against such effects (Schlossmacher et al. 2017). Furthermore, they highlight a potential reason for generally mixed findings in the field of CF-suppressed emotional faces (Pournaghdali and Schwartz 2020). Previous studies often differed in the way they implemented an individual contrast calibration, allowing for stark differences in the contrast used in the nonconscious conditions relative to the individual participants’ awareness threshold. Thus, in some studies, stimulus strength might have been well below the awareness threshold (like in our low-contrast condition) and, in others, very close to it (like in our medium-contrast condition). The fact that the chosen contrast drastically alters (the) ERP effects might explain conflicting results in previous studies. Furthermore, our results highlight that the highest contrast at which stimuli are just suppressed seems to be a desirable target for future studies.

At first glance, our findings would implicate late positivities as an index of conscious emotional differentiation since fearful faces elicited a higher positivity than neutral ones in all conscious conditions. This pattern is in line with the proposition of the global neuronal workspace theory, which links later components to conscious processing (Dehaene and Changeux 2011, Mashour et al. 2020). However, it should be taken into account that in our study, task-related confounds might have influenced the pattern of results (Aru et al. 2012, Tsuchiya et al. 2015). Participants had to answer detection and discrimination questions on a random subset of trials, which might have elicited postperceptual processes like decision-making in the majority of conscious trials. In several studies, it could be shown that prominent late positivities are abolished if stimuli are task-irrelevant and postperceptual processes are impeded (Pitts et al. 2012, Shafto and Pitts 2015, Förster et al. 2020, Schlossmacher et al. 2020, 2021, Dellert et al. 2021, 2022). However, this argumentation leaves open the question of whether there are components that specifically index conscious emotional processing (but see Sun et al. 2023).

There are some limitations in the current study that should be noted. First, fearful and neutral stimuli differed physically. Thus, low-level features inherent to fearful and neutral stimuli might have contributed to the effects we found (Moors et al. 2019). In order to investigate emotion effects independent of potential low-level confounds, future studies could, e.g., use fear conditioning of neutral expressions to circumvent this issue (see, e.g., Morris et al. 1998, Sabatini et al. 2009). Second, a contrast calibration prior to the experiment and block-wise recalibrations can limit the possibility of residual awareness, but cannot fully exclude it. These procedures necessarily rely on performance estimates averaged across several trials, and if performance increased during a block, e.g. due to perceptual learning, we would overestimate the awareness threshold at the end of those blocks. Given that objective performance measures averaged across all trials were at chance level, this overestimation is presumably small, but nevertheless possible. Also, the calibration did not yield a satisfactory result in all participants. *Post hoc* exclusion of participants when inferring nonconscious processing can lead to serious statistical pitfalls (Shanks 2017). While we think that this problem should be considered when interpreting the results, we do not think that effects relied on regression to the mean or residual awareness in the medium-contrast condition for several reasons. Correlating measures of awareness (here detection *d'*) with cluster averages in the medium-contrast condition did not yield significant results but instead evidence for the absence of an effect. Furthermore, conscious and nonconscious conditions varied in their ability to elicit later effects. If the observed early negativity in the medium-contrast condition with CFS solely relied on residual awareness, we would also expect to observe a late positivity, as was the case in all conscious conditions. In contrast, we found evidence for the absence of a late effect in this condition. Despite this, future studies should optimize the contrast calibration in order to be able to include all participants in the analysis. As a third limitation, we did not equate baseline contrast across images prior to the experiment. The stimuli were grayscale versions of images from the Radboud Faces Database, which contains photographs taken under constant lighting conditions (Langner et al. 2010). Thus, while the images are subjectively similar in contrast, they are not identical. It is therefore possible that the behavioral responses obtained in a randomly chosen third of the trials were confounded by differences in baseline image contrast, leading to a small over- or underestimation of the visibility of the image set as a whole. Fourth, we only used CFS to test the threshold account. Thus, it remains unclear whether this finding can be generalized to other “blinding” techniques. Future studies should address this issue by investigating the influence of stimulus strength on emotional processing using different “blinding” methods.

## Conclusion

In summary, the current results demonstrate that differential effects of facial expressions in nonconscious conditions critically depend on stimulus strength. They offer a compelling explanation for diverging findings in the field. An important implication that can be drawn from the present findings is that future research on nonconscious processing under CFS should aim at presenting stimuli with the highest amount of stimulus strength that is just barely suppressed.

## Supplementary Material

niaf001_Supp

## Data Availability

Data and code are available on the Open Science Framework accessible via https://osf.io/bwzsp/.

## References

[R1] Aru J, Bachmann T, Singer W et al. Distilling the neural correlates of consciousness. *Neurosci Biobehav Rev* 2012;36:737–46. doi: 10.1016/j.neubiorev.2011.12.003 22192881

[R2] Axelrod V, Bar M, Rees G. Exploring the unconscious using faces. *Trends Cogn Sci* 2015;19:35–45. doi: 10.1016/j.tics.2014.11.003 25481216

[R3] Brainard DH . The psychophysics toolbox. *Spatial Vision* 1997;10:433–6. doi: 10.1163/156856897X00357 9176952

[R4] Dehaene S, Changeux J-P. Experimental and theoretical approaches to conscious processing. *Neuron* 2011;70:200–27. doi: 10.1016/j.neuron.2011.03.018 21521609

[R5] Dellert T, Krebs S, Bruchmann M et al. Neural correlates of consciousness in an attentional blink paradigm with uncertain target relevance. *NeuroImage* 2022;264C:119679. doi: 10.1016/j.neuroimage.2022.119679 36220535

[R6] Dellert T, Müller-Bardorff M, Schlossmacher I et al. Dissociating the neural correlates of consciousness and task relevance in face perception using simultaneous EEG-fMRI. *J Neurosci* 2021;41:7864–75. doi: 10.1523/JNEUROSCI.2799-20.2021 34301829 PMC8445054

[R7] Doradzińska Ł, Bola M. I focus only when I see your fear—fearful faces are not prioritized by attention when processed outside of awareness. *Cereb Cortex* 2023;33:9233–49. doi: 10.1093/cercor/bhad194 37339886

[R8] Durston AJ, Itier RJ. The early processing of fearful and happy facial expressions is independent of task demands – support from mass univariate analyses. *Brain Res* 2021;1765:147505. doi: 10.1016/j.brainres.2021.147505 33915164

[R9] Eimer M, Holmes A. Event-related brain potential correlates of emotional face processing. *Neuropsychologia* 2007;45:15–31. doi: 10.1016/j.neuropsychologia.2006.04.022 16797614 PMC2383989

[R10] Eimer M, Kiss M, Holmes A. Links between rapid ERP responses to fearful faces and conscious awareness. *J Neuropsychol* 2008;2:165–81. doi: 10.1348/174866407X245411 19330049 PMC2661068

[R11] Eiserbeck A, Enge A, Rabovsky M et al. Distrust before first sight? Examining knowledge- and appearance-based effects of trustworthiness on the visual consciousness of faces. *Conscious Cogn* 2024;117:103629. doi: 10.1016/j.concog.2023.103629 38150782

[R12] Faul F, Erdfelder E, Lang A-G et al. G* Power 3: a flexible statistical power analysis program for the social, behavioral, and biomedical sciences. *Behav Res Methods* 2007;39:175–91. doi: 10.3758/BF03193146 17695343

[R13] Fields EC . Factorial Mass Univariate ERP Toolbox. Computer software, 2017. github.com/ericcfields/FMUT/releases (23 November 2023, date last accessed).

[R14] Fields EC, Kuperberg GR. Having your cake and eating it too: flexibility and power with mass univariate statistics for ERP data. *Psychophysiology* 2020;57:e13468. doi: 10.1111/psyp.13468 PMC7269415 31456213

[R15] Förster J, Koivisto M, Revonsuo A. ERP and MEG correlates of visual consciousness: the second decade. *Conscious Cogn* 2020;80:102917. doi: 10.1016/j.concog.2020.102917 32193077

[R16] Gelbard-Sagiv H, Faivre N, Mudrik L et al. Low-level awareness accompanies “unconscious” high-level processing during continuous flash suppression. *J Vis* 2016;16:3. doi: 10.1167/16.1.3 26756173

[R17] Geng H, Zhang S, Li Q et al. Dissociations of subliminal and supraliminal self-face from other-face processing: behavioral and ERP evidence. *Neuropsychologia* 2012;50:2933–42. doi: 10.1016/j.neuropsychologia.2012.07.040 22898645

[R18] Hajcak G Weinberg A MacNamara A et al. ERPs and the study of emotion. In: Luck SJ and Kappenman ES (eds.), *The Oxford Handbook of Event-related Potential Components*. Oxford, UK: Oxford University Press, 2012, 441–72.

[R19] Hinojosa JA, Mercado F, Carretié L. N170 sensitivity to facial expression: a meta-analysis. *Neurosci Biobehav Rev* 2015;55:498–509. doi: 10.1016/j.neubiorev.2015.06.002 26067902

[R20] Ille N, Berg P, Scherg M. Artifact correction of the ongoing EEG using spatial filters based on artifact and brain signal topographies. *J Clin Neurophysiol* 2002;19:113–24. doi: 10.1097/00004691-200203000-00002 11997722

[R21] Jeffreys H . *Theory of Probability*. UK: Oxford University Press, 1961.

[R22] Jiang Y, Shannon RW, Vizueta N et al. Dynamics of processing invisible faces in the brain: automatic neural encoding of facial expression information. *NeuroImage* 2009;44:1171–7. doi: 10.1016/j.neuroimage.2008.09.038 18976712 PMC3180886

[R23] Kiss M, Eimer M. ERPs reveal subliminal processing of fearful faces. *Psychophysiology* 2008;45:318–26. doi: 10.1111/j.1469-8986.2007.00634.x 17995905 PMC2375009

[R24] Kleiner M, Brainard DH, Pelli DG. What’s new in Psychtoolbox-3? *Perception* 2007;36:14.

[R25] Kouider S, de Gardelle V, Sackur J et al. How rich is consciousness? The partial awareness hypothesis. *Trends Cogn Sci* 2010;14:301–7. doi: 10.1016/j.tics.2010.04.006 20605514

[R26] Kouider S, Dupoux E. Partial awareness creates the “Illusion” of subliminal semantic priming. *Psychol Sci* 2004;15:75–81. doi: 10.1111/j.0963-7214.2004.01502001.x 14738512

[R27] Langner O, Dotsch R, Bijlstra G et al. Presentation and validation of the Radboud Faces Database. *Cognition & Emotion* 2010;24:1377–1388. doi: 10.1080/02699930903485076

[R28] Langner O, Dotsch R, Bijlstra G et al. Presentation and validation of the radboud faces database. *Cogn Emot* 2010;24:1377–88. doi: 10.1080/02699930903485076

[R29] Liddell BJ, Williams LM, Rathjen J et al. A temporal dissociation of subliminal versus supraliminal fear perception: an event-related potential study. *J Cognitive Neurosci* 2004;16:479–86. doi: 10.1162/089892904322926809 15072682

[R30] Luck SJ . *An Introduction to the Event-related Potential Technique*. Cambridge, Massachusetts, USA: MIT Press, 2005.

[R31] Macmillan N, and Creelman CD. *Detection Theory: A User’s Guide*, 2nd edn. New York, NY, USA: Lawrence Erlbaum Associates, 2005.

[R32] Malejka S, Vadillo MA, Dienes Z et al. Correlation analysis to investigate unconscious mental processes: a critical appraisal and mini-tutorial. *Cognition* 2021;212:104667. doi: 10.1016/j.cognition.2021.104667 33975175

[R33] Mashour GA, Roelfsema P, Changeux J-P et al. Conscious processing and the global neuronal workspace hypothesis. *Neuron* 2020;105:776–98. doi: 10.1016/j.neuron.2020.01.026 32135090 PMC8770991

[R34] Merikle PM . Unconscious perception revisited. *Percept Psychophys* 1982;31:298–301. doi: 10.3758/BF03202538 7088675

[R35] Miles WR . Ocular dominance demonstrated by unconscious sighting. *J Exp Psychol* 1929;12:113–26. doi: 10.1037/h0075694

[R36] Moors P, Gayet S, Hedger N et al. Three criteria for evaluating high-level processing in continuous flash suppression. *Trends Cogn Sci* 2019;23:267–9. doi: 10.1016/j.tics.2019.01.008 30795895

[R37] Moors P, Hesselmann G, Wagemans J et al. Continuous flash suppression: stimulus fractionation rather than integration. *Trends Cogn Sci* 2017;21:719–21. doi: 10.1016/j.tics.2017.06.005 28690078

[R38] Morris JS, Öhman A, and Dolan RJ. Conscious and unconscious emotional learning in the human amygdala. *Nature* 1998;393:467–70. doi: 10.1038/30976 9624001

[R39] Mudrik L, Deouell LY. Neuroscientific evidence for processing without awareness. *Annu Rev Neurosci* 2022;45:403–23. doi: 10.1146/annurev-neuro-110920-033151 35803585

[R40] Neath-Tavares KN, Itier RJ. Neural processing of fearful and happy facial expressions during emotion-relevant and emotion-irrelevant tasks: a fixation-to-feature approach. *Biol Psychol* 2016;119:122–40. doi: 10.1016/j.biopsycho.2016.07.013 27430934 PMC5319862

[R41] Pegna AJ, Darque A, Berrut C et al. Early ERP modulation for task-irrelevant subliminal faces. *Front Psychol* 2011;2:1–10. doi: 10.3389/fpsyg.2011.00088 21687457 PMC3110345

[R42] Pegna AJ, Landis T, Khateb A. Electrophysiological evidence for early non-conscious processing of fearful facial expressions. *Int J Psychophysiol* 2008;70:127–36. doi: 10.1016/j.ijpsycho.2008.08.007 18804496

[R43] Pelli DG . The VideoToolbox software for visual psychophysics: transforming numbers into movies. *Spatial Vision* 1997;10:437–42. doi: 10.1163/156856897X00366 9176953

[R44] Peremen Z, Lamy D. Comparing unconscious processing during continuous flash suppression and meta-contrast masking just under the limen of consciousness. *Conscious Res* 2014;5:969. doi: 10.3389/fpsyg.2014.00969 PMC4160875 25309469

[R45] Pitts MA, Martínez A, Hillyard SA. Visual processing of contour patterns under conditions of inattentional blindness. *J Cognitive Neurosci* 2012;24:287–303. doi: 10.1162/jocn_a_00111 21812561

[R46] Porta GD . De refractione optices parte: Libri novem. Apud Io. Iacobum Carlinum & Antonium Pacem, 1593.

[R47] Pournaghdali A, Schwartz BL. Continuous flash suppression: known and unknowns. *Psychonomic Bull Rev* 2020;27:1071–103. doi: 10.3758/s13423-020-01771-2 32671572

[R48] Qiu Z, Becker SI, Pegna AJ. Spatial attention shifting to emotional faces is contingent on awareness and task relevancy. *Cortex* 2022a;151:30–48. doi: 10.1016/j.cortex.2022.02.009 35390549

[R49] Qiu Z, Becker SI, Pegna AJ. The effects of spatial attention focus and visual awareness on the processing of fearful faces: an ERP study. *Brain Sci* 2022b;12:Article7. doi: 10.3390/brainsci12070823 PMC9313043 35884630

[R50] Qiu Z, Jiang J, Becker SI et al. Attentional capture by fearful faces requires consciousness and is modulated by task-relevancy: a dot-probe EEG study. *Front Neurosci* 2023;17:1152220. doi: 10.3389/fnins.2023.1152220 PMC10076762 37034154

[R51] Qiu Z, Lei X, Becker SI et al. Neural activities during the processing of unattended and unseen emotional faces: a voxel-wise meta-analysis. *Brain Imaging Behav* 2022;16:2426–43. doi: 10.1007/s11682-022-00697-8 35739373 PMC9581832

[R52] R Core Team . R: A Language and Environment for Statistical Computing. Software, 2023. http://www.R-project.org/ (13 March 2023, date last accessed).

[R53] Rellecke J, Palazova M, Sommer W et al. On the automaticity of emotion processing in words and faces: event-related brain potentials evidence from a superficial task. *Brain Cogn* 2011;77:23–32. doi: 10.1016/j.bandc.2011.07.001 21794970

[R54] Sabatini E, Penna SD, Franciotti R et al. Brain structures activated by overt and covert emotional visual stimuli. *Brain Res Bull* 2009;79:258–64. doi: 10.1016/j.brainresbull.2009.03.001 19480985

[R55] Schindler S, Bruchmann M, Bublatzky F et al. Modulation of face- and emotion-selective ERPs by the three most common types of face image manipulations. *Soc Cognit Affect Neurosci* 2019;14:493–503. doi: 10.1093/scan/nsz027 30972417 PMC6545565

[R56] Schindler S, Bruchmann M, Gathmann B et al. Effects of low-level visual information and perceptual load on P1 and N170 responses to emotional expressions. *Cortex* 2021a;136:14–27. doi: 10.1016/j.cortex.2020.12.011 33450599

[R57] Schindler S, Bruchmann M, Steinweg A-L et al. Attentional conditions differentially affect early, intermediate and late neural responses to fearful and neutral faces. *Soc Cognit Affect Neurosci* 2020;15:765–74. doi: 10.1093/scan/nsaa098 32701163 PMC7511883

[R58] Schindler S, Bruchmann M, Straube T. Feature-based attention interacts with emotional picture content during mid-latency and late ERP processing stages. *Biol Psychol* 2022a;170:108310. doi: 10.1016/j.biopsycho.2022.108310 35278527

[R59] Schindler S, Bublatzky F. Attention and emotion: an integrative review of emotional face processing as a function of attention. *Cortex* 2020;130:362–86. doi: 10.1016/j.cortex.2020.06.010 32745728

[R60] Schindler S, Heinemann J, Bruchmann M et al. No trait anxiety influences on early and late differential neuronal responses to aversively conditioned faces across three different tasks. *Cognit Affect Behav Neurosci* 2022b;22:1157–71. doi: 10.3758/s13415-022-00998-x 35352267 PMC9458573

[R61] Schindler S, Richter TS, Bruchmann M et al. Effects of task load, spatial attention, and trait anxiety on neuronal responses to fearful and neutral faces. *Psychophysiology* 2022c;59:e14114. doi: 10.1111/psyp.14114 35652518

[R62] Schindler S, Tirloni C, Bruchmann M et al. Face and emotional expression processing under continuous perceptual load tasks: an ERP study. *Biol Psychol* 2021b;161:108056. doi: 10.1016/j.biopsycho.2021.108056 33636248

[R63] Schlossmacher I, Dellert T, Bruchmann M et al. Dissociating neural correlates of consciousness and task relevance during auditory processing. *NeuroImage* 2021;228:117712. doi: 10.1016/j.neuroimage.2020.117712 33387630

[R64] Schlossmacher I, Dellert T, Pitts M et al. Differential effects of awareness and task relevance on early and late ERPs in a no-report visual oddball paradigm. *J Neurosci* 2020;40:2906–13. doi: 10.1523/JNEUROSCI.2077-19.2020 32122954 PMC7117899

[R65] Schlossmacher I, Junghöfer M, Straube T et al. No differential effects to facial expressions under continuous flash suppression: an event-related potentials study. *NeuroImage* 2017;163:276–85. doi: 10.1016/j.neuroimage.2017.09.034 28939431

[R66] Schmidt T, Vorberg D. Criteria for unconscious cognition: three types of dissociation. *Percept Psychophys* 2006;68:489–504. doi: 10.3758/BF03193692 16900839

[R67] Shafto JP, Pitts MA. Neural signatures of conscious face perception in an inattentional blindness paradigm. *J Neurosci* 2015;35:10940–8. doi: 10.1523/JNEUROSCI.0145-15.2015 26245958 PMC6605277

[R68] Shanks DR . Regressive research: the pitfalls of post hoc data selection in the study of unconscious mental processes. *Psychonomic Bull Rev* 2017;24:752–75. doi: 10.3758/s13423-016-1170-y PMC5486877 27753047

[R69] Simpson WA . The method of constant stimuli is efficient. *Percept Psychophys* 1988;44:433–6. doi: 10.3758/BF03210427 3226892

[R70] Smith ML . Rapid processing of emotional expressions without conscious awareness. *Cereb Cortex* 2012;22:1748–60. doi: 10.1093/cercor/bhr250 21955918

[R71] Stein T . The breaking continuous flash suppression paradigm. In: Hesselmann G (ed.), *Transitions between Consciousness and Unconsciousness*, 1st edn. New York, NY, USA: Routledge, 2019, 1–38.

[R72] Sterzer P, Stein T, Ludwig K et al. Neural processing of visual information under interocular suppression: a critical review. *Front Psychol* 2014;5:453. doi: 10.3389/fpsyg.2014.00453 PMC4032950 24904469

[R73] Sun B, Zeng X, Chen X et al. Neural correlates of conscious processing of emotional faces: evidence from event-related potentials. *Neuropsychologia* 2023;182:108478. doi: 10.1016/j.neuropsychologia.2023.108478 36707025

[R74] Tamietto M, de Gelder B. Neural bases of the non-conscious perception of emotional signals. *Nat Rev Neurosci* 2010;11:697–709. doi: 10.1038/nrn2889 20811475

[R75] Tsuchiya N, Koch C. Continuous flash suppression reduces negative afterimages. *Nat Neurosci* 2005;8:1096–101. doi: 10.1038/nn1500 15995700

[R76] Tsuchiya N, Wilke M, Frässle S et al. No-report paradigms: extracting the true neural correlates of consciousness. *Trends Cogn Sci* 2015;19:757–70. doi: 10.1016/j.tics.2015.10.002 26585549

[R77] Walentowska W, Wronka E. Trait anxiety and involuntary processing of facial emotions. *Int J Psychophysiol* 2012;85:27–36. doi: 10.1016/j.ijpsycho.2011.12.004 22210124

[R78] Wieser MJ, Gerdes ABM, Greiner R et al. Tonic pain grabs attention, but leaves the processing of facial expressions intact—evidence from event-related brain potentials. *Biol Psychol* 2012;90:242–8. doi: 10.1016/j.biopsycho.2012.03.019 22503790

[R79] Yang E, Blake R, McDonald JE. A new interocular suppression technique for measuring sensory eye dominance. *Invest Ophthalmol Vis Sci* 2010;51:588. doi: 10.1167/iovs.08-3076 PMC2810859 19628736

[R80] Yaron I, Zeevi Y, Korisky U et al. Progressing, not regressing: a possible solution to the problem of regression to the mean in unconscious processing studies. *Psychonomic Bull Rev* 2024;31:49–64. doi: 10.3758/s13423-023-02326-x PMC10867080 37528278

[R81] Zotto MD, Pegna AJ. Processing of masked and unmasked emotional faces under different attentional conditions: an electrophysiological investigation. *Front Psychol* 2015;6:1691. doi: 10.3389/fpsyg.2015.01691 PMC4628105 26583003

