# Supplementary material for: The influence of signal strength on conscious and nonconscious neural processing of emotional faces
Source: Neurosci Conscious. 2025 Feb 5;2025(1):niaf001. doi: 10.1093/nc/niaf001 (PMC11799861; doi:10.1093/nc/niaf001)
Supplement: niaf001_Supp [file niaf001_supp.zip › suppl_data/Supplement.docx]

**Supplement**

***Late positivities.*** Without CFS, cluster-based permutations revealed a significant main effect of contrast (sum(*F*) = 34680.10 , *p* < .001) including time points from 300 to 800 ms with a temporal peak at 324 ms. Furthermore, a significant main effect of facial expression (sum(*F*) = 5442.89, *p* = .001) extending from 300 to 800 ms with a temporal peak at 564 ms was found. No significant interaction was found (all cluster *p*s > .26). Further inspection of the main effect of facial expression revealed a stronger positivity for fearful compared to neutral faces.

With CFS, cluster-based permutations revealed a significant main effect of contrast (sum(*F*) = 46548.12, *p* < .001) including time points from 300 to 800 ms with a temporal peak at 428 ms. No significant main effect of facial expression (all cluster *p*s > .10) and no significant interaction (all cluster *p*s > .38) was found. As neither the main effect of facial expression nor the interaction revealed significant clusters, we wanted to investigate whether we could obtain evidence for the absence of an effect. Therefore, we extracted cluster averages based on the significant main effect of facial expression in the condition without CFS. Testing these cluster averages against zero revealed evidence for the absence of an effect in the low and medium contrast conditions (low: *t*(47) = 0.20, *p_holm_* = 1, BF_01_ = 6.26; medium: *t*(47) = -0.18, *p_holm_* = 1, BF_01_ = 6.27), while in the high contrast condition fearful expressions elicited a stronger positivity than neutral expressions (*t*(47) = 2.54, *p_holm_* = .01, BF_10_ = 7.36).
